# FTI-277 inhibits smooth muscle cell calcification by up-regulating PI3K/Akt signaling and inhibiting apoptosis

**DOI:** 10.1371/journal.pone.0196232

**Published:** 2018-04-24

**Authors:** Arvind Ponnusamy, Smeeta Sinha, Gareth D. Hyde, Samantha J. Borland, Rebecca F. Taylor, Emma Pond, Heather J. Eyre, Colette A. Inkson, Andrew Gilmore, Nick Ashton, Philip A. Kalra, Ann E. Canfield

**Affiliations:** 1 Vascular Research Group, Salford Royal NHS Foundation Trust, Salford, United Kingdom; 2 Division of Cardiovascular Sciences, School of Medical Sciences, Faculty of Biology, Medicine and Health, Manchester Academic Health Sciences Centre, University of Manchester, Manchester, United Kingdom; 3 Division of Cancer Studies & Wellcome Trust Centre for Cell-Matrix Research, School of Medical Sciences, Faculty of Biology, Medicine and Health, Manchester Academic Health Sciences Centre, University of Manchester, Manchester, United Kingdom; University of California, Los Angeles, UNITED STATES

## Abstract

**Background:**

Vascular calcification is associated with increased cardiovascular morbidity and mortality in patients with atherosclerosis, diabetes and chronic kidney disease. However, no viable treatments for this condition have been identified. This study aimed to determine whether farnesyl transferase inhibitors (FTIs) can reduce vascular calcification and the mechanism by which this reduction occurs.

**Results:**

We demonstrate that FTI-277 significantly inhibits phosphate-induced mineral deposition by vascular smooth muscle cells (VSMC) *in vitro*, prevents VSMC osteogenic differentiation, and increases mRNA expression of matrix Gla protein (MGP), an inhibitor of mineralization. FTI-277 increases Akt signaling in VSMC in short-term serum-stimulation assays and in long-term mineralization assays. In contrast, manumycin A has no effect on Akt signaling or mineralization. Co-incubation of VSMC with FTI-277 and SH6 (an Akt inhibitor) significantly reduces the inhibitory effect of FTI-277 on mineralization, demonstrating that FTI-277 inhibits calcification by activating Akt signaling. Over-expression of the constitutively active p110 sub-unit of PI3K in VSMC using adenovirus activates Akt, inhibits mineralization, suppresses VSMC differentiation and significantly enhances MGP mRNA expression. FTI-277 also inhibits phosphate-induced activation of caspase 3 and apoptosis of VSMC, and these effects are negated by co-incubation with SH6. Finally, using an *ex vivo* model of vascular calcification, we demonstrate that FTI-277 inhibits high phosphate-induced mineralization in aortic rings derived from rats with end-stage renal failure.

**Conclusions:**

Together, these results demonstrate that FTI-277 inhibits VSMC mineral deposition by up-regulating PI3K/Akt signaling and preventing apoptosis, suggesting that targeting farnesylation, or Akt specifically, may have therapeutic potential for the prevention of vascular calcification.

## Introduction

Vascular calcification is associated with increased cardiovascular morbidity and mortality in patients with atherosclerosis, diabetes and chronic kidney disease (CKD) [1–3]. It is a complex process involving aberrant mineral metabolism, dysregulation of naturally occurring inhibitors of calcification (e.g. matrix Gla protein [MGP]), osteo/chondrogenic differentiation of vascular smooth muscle cells (VSMC), VSMC apoptosis, and the release of matrix vesicles [4–6]. However, viable therapeutic approaches to target vascular calcification are limited.

Many cellular processes involved in vascular calcification are regulated by protein prenylation [7]. Farnesyl transferase catalyzes the transfer of a 15 carbon isoprenoid lipid from farnesyl diphosphate onto a cysteine residue in the C-terminal CAAX box of several proteins, including small GTPases (Ras, RhoB) and prelamin A. Without this post-translational modification, these proteins cannot exert their normal cellular functions [7]. Drugs that target protein farnesylation (farnesyl transferase inhibitors, FTIs) were initially developed to inhibit oncogenic Ras [8, 9] However, there is now increasing interest in the therapeutic potential of FTIs in other clinical settings, although conflicting data have been obtained. For example, inhibiting farnesylation using manumycin A prevented the development of atherosclerosis in the aortic sinus of ApoE-null mice *in vivo* [10]. The farnesyl transferase inhibitor, R115777, has also been shown to be protective against atherosclerosis and calcification in uraemic ApoE-null mice; however, this drug had no effect on atherosclerosis in the absence of uraemia [11]. FTIs have also been shown to alleviate the vascular phenotypes in mouse models of progeria [12] and clinical trials for children with Hutchinson-Gilford progeria syndrome have shown that the FTI, lonafarnib, improves vascular stiffness, bone structure, audiological status and survival [13, 14].

Although these studies have highlighted the potential of targeting farnesylation as a therapeutic option for cardiovascular disease, some important questions remain to be resolved. For example, do these drugs have any effect on calcification in the absence of atherosclerosis and how do these drugs exert their effects? Therefore, as part of our strategy to identify potential therapeutic interventions for vascular calcification, we investigated whether FTIs can regulate the deposition of a mineralized matrix by VSMC *in vitro* and *ex vivo* in the absence of atherosclerosis using aortic rings from rats with end stage renal failure. We used two different inhibitors, FTI-277 and manumycin A, as they target farnesylation in different ways: FTI-277 is a CAAX peptidomimetic which competes with target proteins for the binding of farnesyl residues, whereas manumycin A is a farnesyl pyrophosphate analogue which competes with farnesyl pyrophosphate at the farnesyl transferase binding site. We also examined the mechanism(s) by which these FTIs exert their effects in VSMC.

This study demonstrates for the first time that FTI-277, but not manumycin A, significantly inhibits the deposition of a mineralized matrix by VSMC, and that activation of Akt signaling by FTI-277 is essential for this inhibition. We also show that FTI-277 inhibits high phosphate-induced mineralization in aortic rings derived from rats in end stage renal failure. This study, and the fact that clinical trials show that FTIs are relatively well-tolerated in humans [13–16], suggests that specific FTIs may have therapeutic potential for vascular calcification even in the absence of atherosclerosis.

## Materials and methods

### Reagents

All reagents were analytical grade and obtained from Sigma-Aldrich (UK) unless otherwise stated. FTI-277, manumycin A and SH6 were solubilized in DMSO; an equivalent volume of DMSO was added as a vehicle control in experiments. Antibodies to active caspase 3 (#9661), total caspase 3 (#9662), phospho-Akt^ser473^ (#4060) and total Akt (#9272) were from Cell Signaling (USA). The Active Ras Pull-Down and Detection kit (#89855D) was from Pierce / Thermo Scientific.

### Cell culture

VSMC were isolated from bovine aortic explants obtained from a local abattoir (J&B Fitton Ltd, Shaw, Oldham, UK) and routinely cultured in high glucose Dulbecco’s Modified Eagle Medium (DMEM) supplemented with 2 mM L-glutamine, 100 U/ml penicillin, 1.4 μM streptomycin, 1 mM sodium pyruvate, 1x non-essential amino acids and 10% (v/v) fetal calf serum (FCS) (referred to as 10% FCS-DMEM). Different preparations of VSMC from two animals were used for experiments; cells were used between passage 8 and 16. In some experiments, VSMC were infected at 90% confluence using recombinant adenovirus encoding the constitutively active (CA)-p110α sub-unit of PI3K (pacAd5CMVPI3K) or empty vector (Ad5/*BgI*II) recombinant adenovirus as a control [17, 18]. The CA-p110α sub-unit of PI3K contained a myc-tag and a prenylation signal (CAAX) [19]. A dose response of adenovirus infection was performed using a range of multiplicity of infection (MOI; 0 to 500) to assess the most appropriate MOI to achieve >90% infection without any cell toxicity. A MOI of 200 was chosen for all experiments. Adenoviral vectors were generated by the Gene Transfer Core (University of Iowa) under the direction of Dr Beverly Davidson.

Human coronary artery VSMC (Invitrogen, Lot # 642644) were maintained in Medium 231 (M-231-500) and smooth muscle growth supplement (S-007-25) (both from Invitrogen). Cells were used between passage 6 and 8 for experiments.

### Mineralization and differentiation assays

VSMC were plated in 6-well plates at 2 x 10^4^ cells / cm^2^ and maintained in 10% FCS-DMEM until confluent (day 0). At this point, the cells were cultured in 10% FCS-DMEM containing β-glycerophosphate (βGP, 3–5 mM [17, 18, 20, 21]) and either FTI-277 (1–20 μM), manumycin A (10 or 20 μM), SH6 (Akt inhibitor, 10 μM) or combinations thereof for up to 12 days; control cells were incubated with βGP but with vehicle. Additional controls included cells incubated in 10% FCS-DMEM plus vehicle but without βGP. In some experiments, FTI-277 was added at specific time points (2–7 days) after the addition of βGP. In experiments where cells were infected with pacAd5CMVPI3K or Ad5/*Bgl*II, the cells were induced to mineralize using βGP as detailed above; and were re-infected with virus 3 and 6 days later [17, 18]. Mineralization was assessed using alizarin red staining; images were captured using a digital camera and analyzed using AnalySIS software (Soft Imaging System, Germany). Mineralization was quantified by eluting the alizarin red dye as previously described [17, 18, 20, 21].

To analyze differentiation, RNA was collected at the time-points specified in the legends using a Qiashredder and RNeasy Mini Kits (Qiagen, UK) and incubated with DNase (Ambion, Life Technologies, UK) to remove any contaminating genomic DNA. cDNA was generated from RNA using TaqMan reverse Transcription Reagents (Invitrogen, Life Technologies, UK), and real-time quantitative PCR (qPCR) for osteogenic markers (Runx2, Msx2), a smooth muscle cell markers (αSMA) and an inhibitor of mineralization (MGP) performed using SYBRgreen (Applied Biosystems, Life Technologies) and an ABI Prism 700 sequence detection system (Applied Biosystems, Life Technologies). Primers were designed using Primer3 (JustBio) and are listed in S1 Table. Results were normalized to two housekeeping genes, RPL12 and PPIA. qPCR reactions were performed in duplicate or triplicate and the data averaged to produce one data-point. The expression of each gene relative to the ratio of two housekeeping genes (PP1A and RPL12, Primer Design, UK) was calculated using the comparative C_t_ method (2^-ΔCt^).

### Apoptosis assays

Human VSMC were plated at 2 x 10^4^ cells / cm^2^ and incubated in Medium 231 containing smooth muscle growth supplement overnight. The cells were washed three times with PBS and then incubated in serum-free DMEM and FTI-277 (10 μM) ± SH6 (10 μM) for 15 minutes. Control cells were incubated in serum-free DMEM and vehicle. To induce apoptosis, the phosphate concentration was increased to 2.6 mM. Two and four hours later, cells were washed twice in PBS, incubated in lysis buffer (20 mM Tris pH 7.6, 150 mM sodium chloride, 1% (v/v) Igepal, 50 mM sodium fluoride, 1 mM EDTA, 1x protease inhibitor cocktail, 1 mM sodium orthovanadate, 1 mM sodium pyrophosphate) for two minutes and cell lysates were collected for analysis of total caspase 3 and active caspase 3 by western blotting (see S2 Table for antibody details). In other experiments, cells were fixed after 12 hours in 4% (v/v) formaldehyde in phosphate buffered saline (PBS) before mounting in Vectorshield containing DAPI (Vector Laboratories, UK). Apoptotic cells (defined as cells with condensed or fragmented nuclei) were counted in a blinded manner and results expressed as percentage of total cells.

### Ras activation assay

VSMC were plated at 1 x 10^4^ cells / cm^2^ in 75 cm^2^ flasks and maintained in 10% FCS-DMEM until 80–90% confluent and then incubated with FTI-277 (10 μM), manumycin A (10 μM), or vehicle control in FCS-DMEM for 72 hours. Cells were serum-starved for 2 hours ± the supplements detailed above or vehicle control, and then incubated in 10% FCS-DMEM for 5 mins. Ras activation was assessed using an Active Ras Pull-Down and Detection Kit, and western blotting according to the manufacturer’s instructions.

### Analysis of Akt signaling

For short-term signaling assays, bovine VSMC were plated at 2 x 10^4^ cells / cm^2^ in 6-well plates and maintained in 10% FCS-DMEM until 80–90% confluent. VSMC were then incubated in 10% FCS-DMEM with FTI-277 (10 μM), manumycin A (10 μM), or DMSO (control) for 77 hours. Cells were washed and incubated with serum-free DMEM for 2 hours ± the supplements detailed above or vehicle, and then stimulated with 10% FCS-DMEM for 5 minutes. Cell lysates were harvested as described above (see apoptosis assays) and analyzed for phospho-Akt^Ser473^ and total Akt by western blotting (see S2 Table for antibody details). In other experiments, confluent VSMC, VSMC transduced with CA-p110α sub-unit of PI3K or VSMC transduced with empty vector (Ad5/*Bgl*ll) recombinant adenovirus were incubated in control medium, βGP-medium, or βGP-medium plus FTI-277 (10 μM), FTI-277 (10 μM) + SH6 (10 μM) or DMSO for 8–11 days. Cell lysates were collected and analyzed for Akt phosphorylation by western blotting, as described above.

### Rat model of CKD

Experiments were performed in accordance with the UK Animals (Scientific Procedures) Act 1986 under the authority of project licence PPL 40/3438. The protocol was approved by the Animal Ethical Review Group of the University of Manchester. All efforts were made to minimize suffering.

Male Sprague-Dawley rats (200-250g Charles River UK Ltd, Kent, UK) were subjected to a two-stage subtotal nephrectomy (SNx, n = 3) under isoflurane anesthesia (4% in O_2_, 2 litres/min) with buprenorphine (0.06 mg, subcutaneous injection) analgesia. Partial nephrectomy of the left kidney was followed one week later by complete removal of the right kidney (75% reduction in renal mass overall) [22]. Sham-operated controls (n = 3) also received two surgical procedures, one week apart, in which the kidneys were exteriorized and returned to the abdominal cavity.

Body weight was recorded weekly; systolic blood pressure was measured in conscious rats using a non-invasive tail-cuff method every 2–3 weeks. Urinary albumin:creatinine ratios were determined every 2 weeks. SNx animals developed end stage renal failure 29–30 weeks after surgery (as judged by progressive increases in systolic blood pressure to >150 mmHg, a urinary albumin:creatinine ratio > 1.0 mg/μmol and rapid weight loss (>20% body weight) over 24–48 hours), at which time they were killed by exsanguination under deep isoflurane anesthesia; age-matched shams were killed at the same time. Terminal urine samples were collected from the bladder for the determination of urinary albumin:creatinine ratio; terminal blood samples were collected to determine creatinine concentration and blood urea nitrogen levels. Urinary albumin concentration was determined using an ELISA (Rat Albumin ELISA Quantitation Set E110-125, Bethyl Laboratories, USA); urine and plasma creatinine concentrations were determined using a standard colorimetric assay (Creatinine Urinary Detection Kit K002, Arbor Assays, USA); blood urea nitrogen was determined using a standard colorimetric assay (Urea Nitrogen Test (Enzymatic-Endpoint) cat. no. 2050–450, Stanbio Laboratory, USA), each according to the manufacturer’s instructions. The results are shown in S3 Table.

### Ex vivo aortic ring assay

Rat aortic rings (3–4 mm) were incubated in serum-free DMEM containing 3.75 U/ml alkaline phosphatase (Promega) and 3.3 mM phosphate ± FTI-277 (10 μM) as described [23]. Control rings were incubated in serum-free DMEM and vehicle. Medium was changed every 48 hours for 10 days. After this time, some rings were incubated in two changes of aortic wash buffer [24] (100 mM CaCl_2_, 20 mM Hepes pH 7.4, 150 mM NaCl and 0.02% (w/v) NaN_3_) at 37°C for 48 hours, blotted dry and weighed. Calcium was extracted using 0.6 M hydrochloric acid for 1 hour and the concentration determined using the *o*-cresolphthalein complexone method [25] and normalized to weight. Other rings were processed for histology, stained with alizarin red and imaged using a Panoramic 250 Flash slide scanner (3D HISTECH).

## Statistics

Data are presented as means ± standard error of the mean (SEM). Data were normalized using log_10_ where required, and analyzed using one-way ANOVA with Tukey post-hoc tests. Differences were accepted as statistically significant at p<0.05.

## Results

### FTI-277 inhibits the osteogenic differentiation of VSMC and the deposition of a mineralized matrix

**Fig 1:**
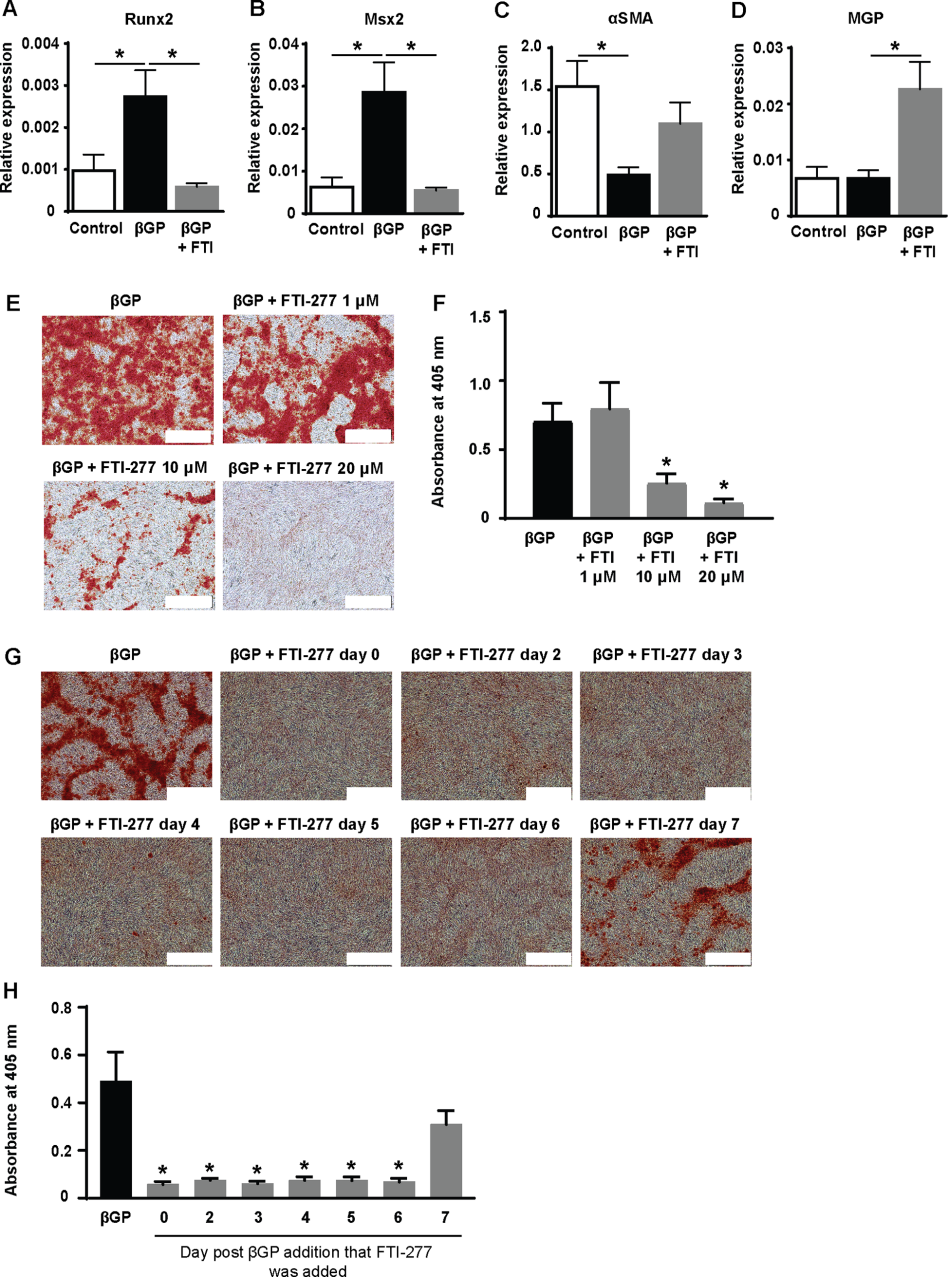
FTI-277 inhibits osteogenic differentiation and mineralization of VSMC. Confluent VSMC were incubated in 10% FCS-DMEM containing βGP (5 mM) and DMSO (1:500) or FTI-277 (20 μM) for 8–10 days. Control cells were incubated in the absence of βGP. RNA was prepared and qPCR for (**A**) Runx2 (n = 11), (**B**) Msx2 (n = 11), (**C**) α-smooth muscle actin (αSMA) (n = 6), and (**D**) matrix Gla protein (MGP) (n = 12) performed. mRNA expression is shown relative to housekeeping genes, PP1A and RPL12. Data are shown as mean ± SEM and were analyzed using one-way ANOVA and Tukey post-hoc tests. *p<0.05 compared to βGP. (**E,F**) Confluent VSMC were incubated in 10% FCS-DMEM containing βGP (5 mM) and DMSO (1:500) or FTI-277 (1, 10, 20 μM) and stained with alizarin red after 10 days. (**E**) Representative phase contrast images; white scale bar = 500 μm. (**F**) Quantification of mineralization (mean ± SEM; n = 19, βGP; n = 9, βGP + 1 μM FTI-277; n = 15, βGP + 10 μM FTI-277; n = 13, βGP + 20 μM FTI-277). Data were normalized using log_10_ and analyzed using one-way ANOVA and Tukey post-hoc tests; *p<0.05 compared to βGP. (**G,H**) Confluent VSMCs were incubated in 10% FCS-DMEM containing βGP (5 mM) and DMSO (1:1000); FTI-277 (10 μM) was added on day 0, 2, 3, 4, 5, 6 or 7. Cells were stained with alizarin red after 8 days. (**G**) Representative phase contrast images, white scale bar = 500 μm. (**H**) Quantification of mineralization (mean ± SEM; n = 6). Data were normalized using log_10_ and analyzed using one-way ANOVA and Tukey post-hoc tests; *p<0.05 compared to βGP.

First, we wanted to check that FTI-277 would inhibit farnesylation in VSMC. We did this using active Ras pull-down assays as decreased Ras activation is a well-accepted read-out for this inhibitor (S1 Fig). Next, we investigated the effects of FTI-277 on the osteogenic differentiation and mineralization of VSMC by using a well-established *in vitro* model of vascular calcification in which confluent cells are incubated in the presence of βGP [17, 18, 20, 21]. These studies demonstrated that FTI-277 prevents the βGP-induced increase in the mRNA expression of the osteogenic transcription factors Runx2 (Fig 1A) and Msx2 (Fig 1B*)*, reduces the βGP-induced decrease in αSMA mRNA expression (Fig 1C) and significantly increases the mRNA levels of matrix Gla protein (an inhibitor of mineralization [26]) (Fig 1D). FTI-277 also significantly inhibits βGP-induced mineral deposition by VSMC and this inhibition is dose-dependent (Fig 1E and 1F). The addition of FTI-277 at later time-points in the mineralization assay (i.e. up to 6 days after βGP was first added) also significantly inhibited βGP-induced mineralization (Fig 1G and 1H).

### FTI-277 inhibits mineralization by activating PI3K/Akt signaling

**Fig 2:**
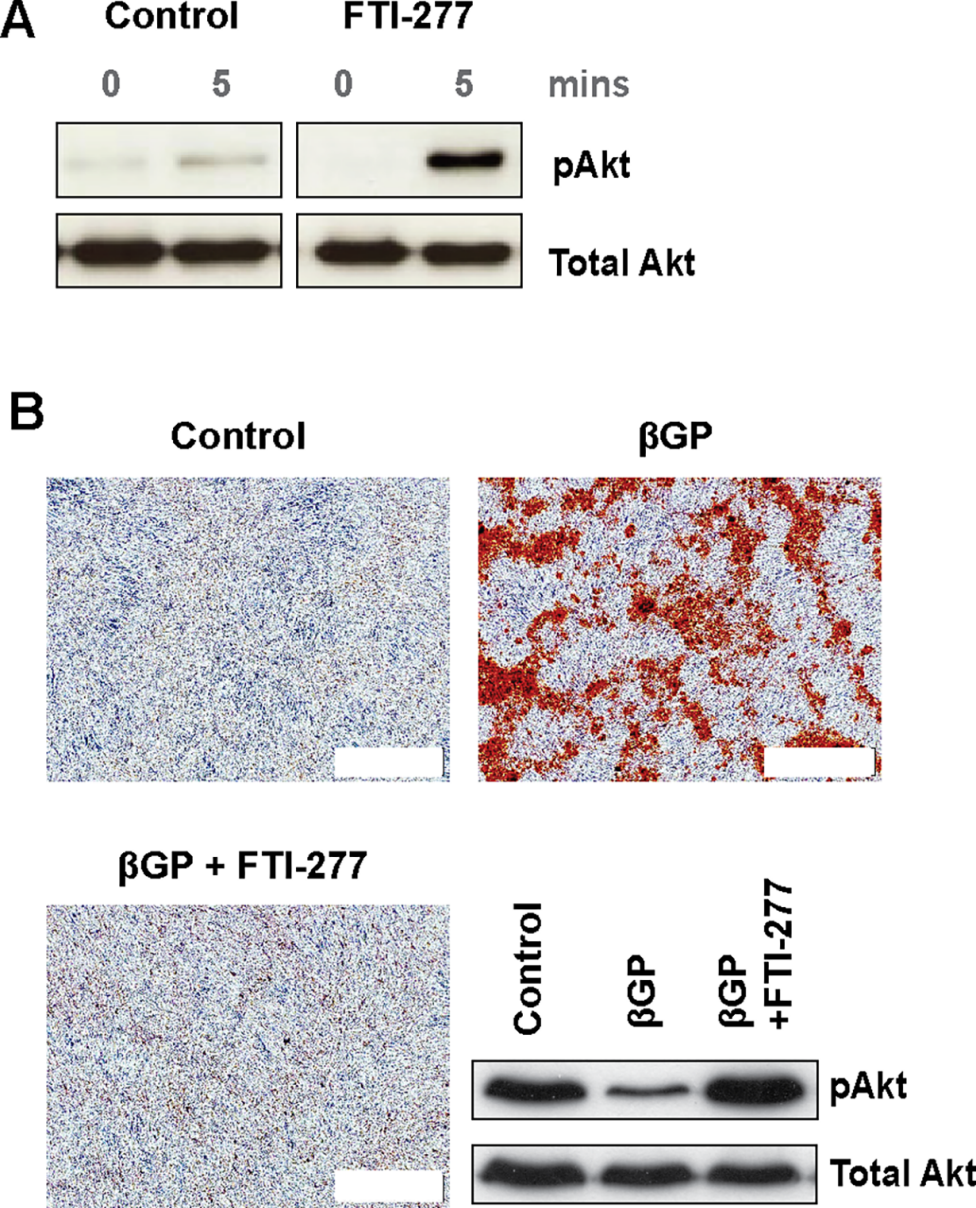
FTI-277 induces Akt signaling in VSMC. (**A**) VSMCs were incubated in 10% FCS-DMEM ± FTI-277 (10 μM) for 77 hours, in serum-free medium ± FTI-277 for 2 hours, and 10% FCS-DMEM for 5 minutes. Cell lysates were collected and analyzed for pAkt and total Akt by western blotting. (**B**) VSMCs were incubated in 10% FCS-DMEM containing DMSO (1:1000), or βGP (5 mM) ± FTI-277 (10 μM) for 10 days. Representative phase contrast images of alizarin red stained cells (white scale bar = 500 μm) and western blots of cell lysates for pAkt and total Akt are shown.

Previous studies have shown that FTI-277 regulates Akt signaling in a cell type- and context-dependent manner [27–34]. The Akt signaling pathway also regulates vascular calcification [17, 35–42]. Therefore, to determine the mechanism by which FTI-277 exerts its effects, we investigated whether VSMC PI3K/Akt signaling is modulated in response to this inhibitor. VSMC were pre-incubated with FTI-277 and serum-starved before stimulation with 10% FCS-DMEM. We showed that FTI-277 markedly increases Akt phosphorylation in response to serum (Fig 2A). FTI-277 also prevented the βGP-induced decrease in Akt phosphorylation during VSMC mineralization (Fig 2B). Interestingly, another farnesyl transferase inhibitor, manumycin A, also inhibited Ras activation (S1 Fig*)*, but did not increase Akt phosphorylation or inhibit βGP-induced mineral deposition by VSMC (S2 Fig). Together, these results suggest that FTI-277-induced Akt phosphorylation in VSMC is important for the inhibition of mineral deposition by this drug.

**Fig 3:**
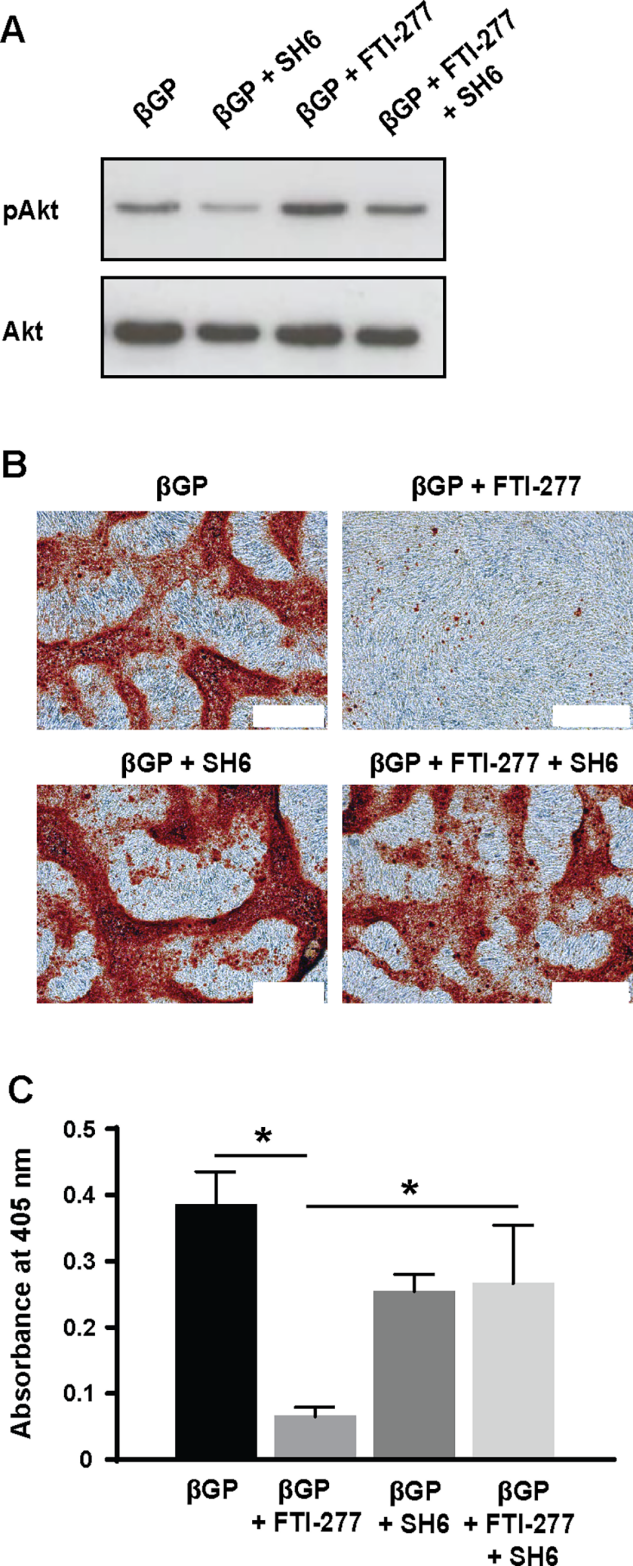
FTI-277 induced Akt signaling is required for inhibition of VSMC mineralization. VSMCs were incubated in 10% FCS-DMEM containing βGP (5 mM) and DMSO (1:1000), FTI-277 (20 μM), SH6 (10 μM), or FTI-277 plus SH6 for up to 9 days, and (**A**) analyzed for pAkt and total Akt by western blotting or (**B,C**) stained with alizarin red. (**B**) Representative phase contrast images of alizarin red-stained cells; white scale bar = 500 μm. (**C**) Quantification of mineralization (mean ± SEM; n = 4). Data were normalized using log_10_ and analyzed using one-way ANOVA and Tukey post-hoc tests; * p<0.05.

To determine whether activation of down-stream Akt signaling is required for the inhibition of mineral deposition by FTI-277, VSMC were incubated in βGP-medium in the presence or absence of FTI-277 and SH6 (a selective Akt inhibitor). We demonstrated that SH6 reduces Akt phosphorylation in VSMC and suppresses the up-regulation in Akt-phosphorylation by FTI-277 (Fig 3A). SH6 also prevented the inhibition of βGP- induced mineralization by FTI-277 (Fig 3B and 3C).

**Fig 4:**
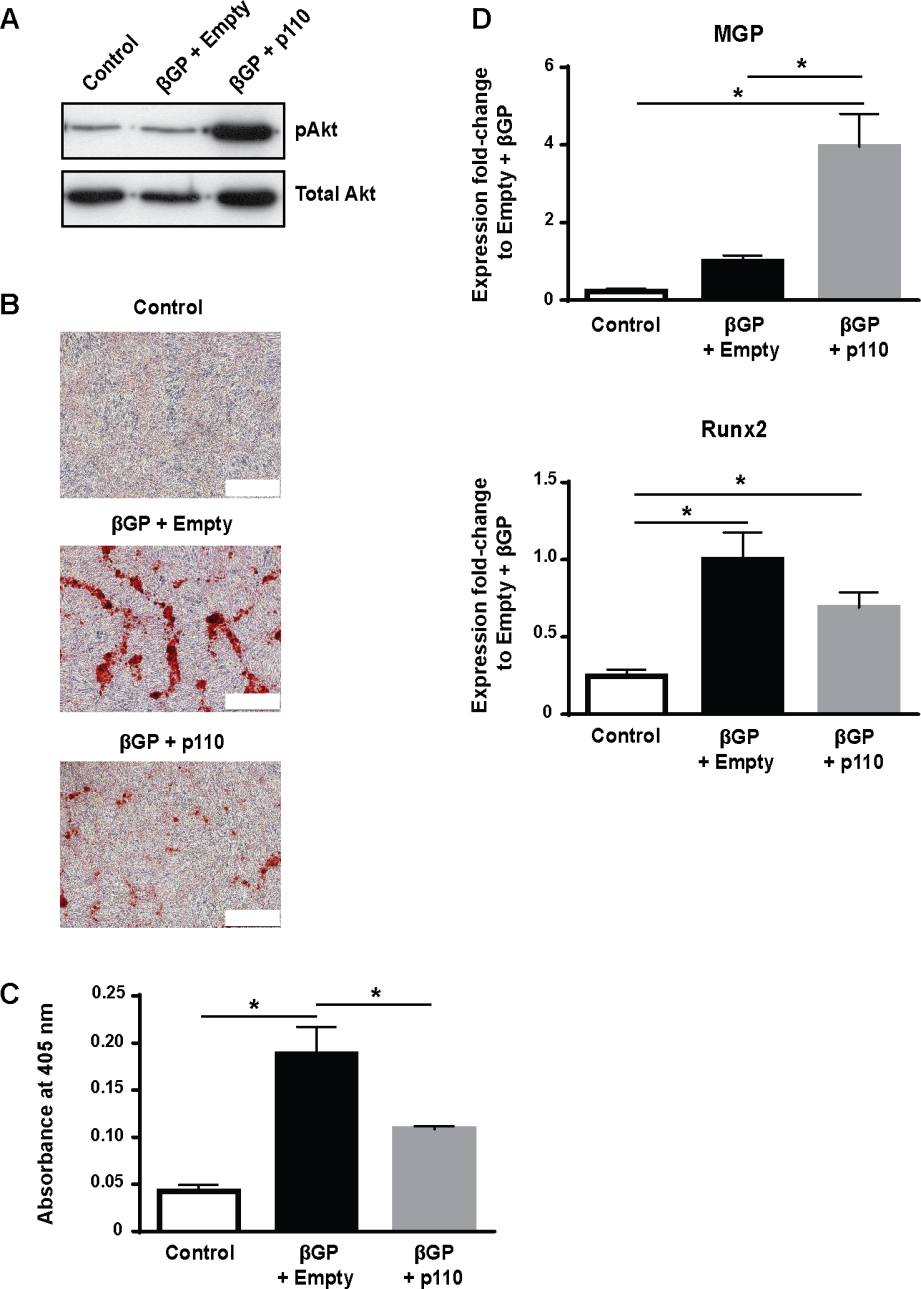
Over-expression of constitutively activated p110 sub-unit of PI3K inhibits mineralization. Constitutively active p110 sub-unit of PI3K (p110) or empty virus (Empty) were over-expressed in VSMC using adenovirus and cells were incubated in 10% FCS-DMEM containing βGP (5 mM) for 9 days. Controls were incubated without BGP. (**A**) Representative western blots of cell lysates for pAkt and total Akt. (**B**) Representative phase contrast images of alizarin red-stained cells (bar = 500 μm). (**C**) Quantification of mineralization (mean ± SEM; n = 6). Data were normalized using log_10_ and analyzed using one-way ANOVA and Tukey post-hoc tests; *p<0.05 compared to βGP. (**D**) qPCR for Runx2 and matrix Gla protein (MGP) was performed. Relative mRNA expression of Runx2 and MGP are shown as fold-change to cells treated with empty virus and βGP (mean ± SEM; n = 6). Data were analyzed using one-way ANOVA and Tukey post-hoc tests; *p<0.05.

To further investigate the role of PI3K/Akt signaling in the inhibition of mineral deposition by VSMC was demonstrated by over-expressing the CA-p110 catalytic subunit of PI3K in VSMC using adenoviruses. These studies confirmed that maintaining high levels of PI3K activates down-stream Akt signaling (Fig 4A) and significantly inhibits βGP-induced mineralization of VSMC (Fig 4B and 4C). Over-expressing the CA-p110 subunit of PI3K in these cells also significantly enhanced MGP mRNA expression compared to empty vector controls incubated with βGP (Fig 4D). It also appeared to reduce Runx2 expression compared to empty vector controls incubated with βGP, although this effect was not significant (Fig 4D). Together, these results demonstrate that FTI-277 prevents osteogenic differentiation and mineralization of VSMC by activating PI3K/Akt signaling.

### FTI-277 inhibits phosphate-induced apoptosis

**Fig 5:**
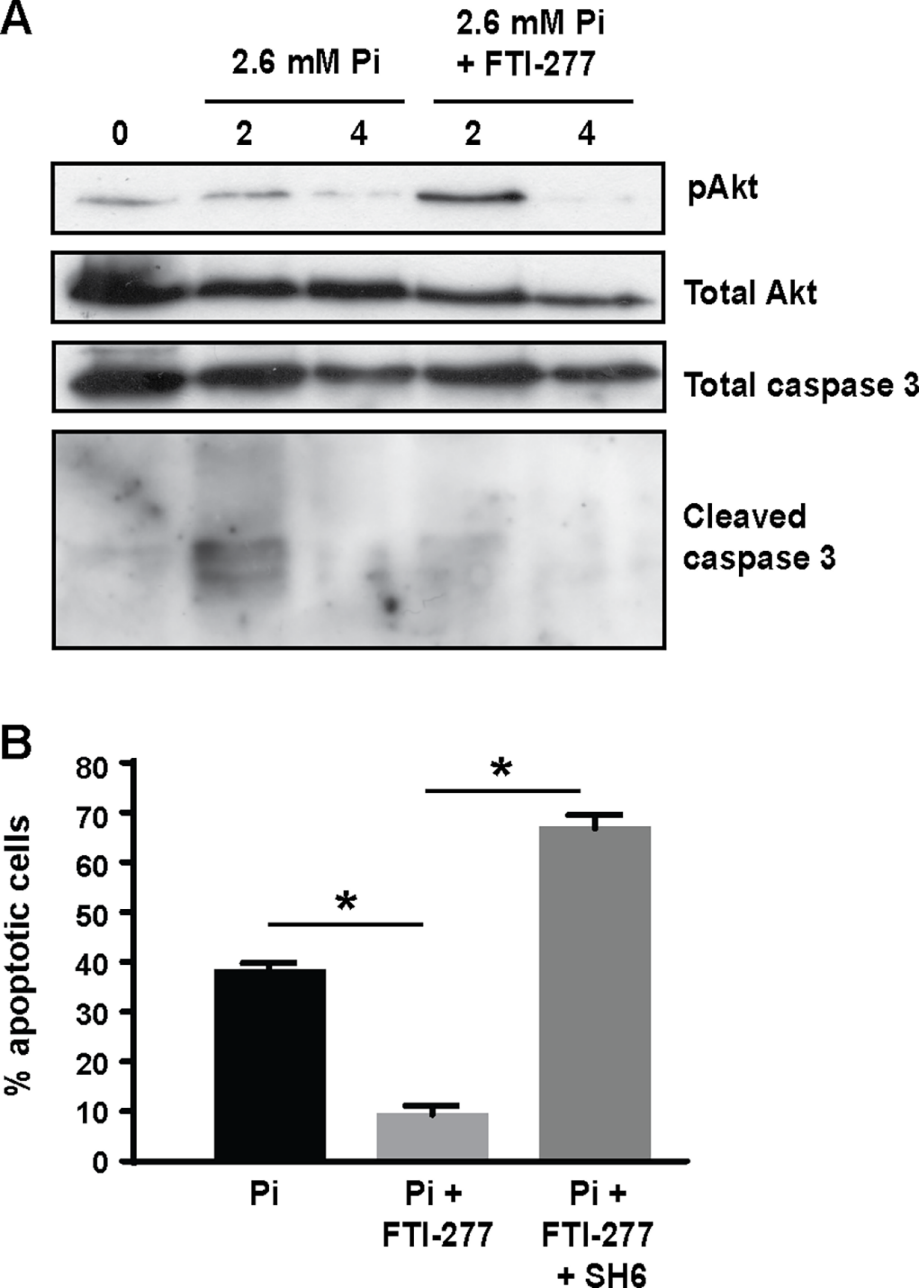
FTI-277 inhibits phosphate-induced apoptosis. **A**) Human VSMCs were incubated in serum-free medium containing elevated phosphate (2.6 mM) ± FTI-277 (10 μM) or vehicle control. Cell lysates were collected 2 and 4 hours later and analysed by western blotting. (**B**) Human VSMCs incubated in serum-free medium containing phosphate (2.6 mM) ± FTI-277 (10 μM) ± SH6 (10 μM) for 12 hours were fixed and stained with DAPI. Apoptotic cells are expressed as a percentage of total cells (mean ± SEM); >750 cells were counted per variable. Data were analyzed by one-way ANOVA and Tukey post-hoc tests; *p<0.05.

Apoptosis of VSMC promotes vascular calcification [17, 43, 44], and PI3K/Akt signaling inhibits apoptosis [45]. Although apoptosis can be detected in VSMCs cultured in calcification medium (which contains serum) for extended periods of time, it is difficult to quantify the level of apoptosis accurately because the cells are variously undergoing differentiation, proliferation and/or apoptosis. Therefore, we induced VSMC apoptosis by culturing human VSMCs in serum-free medium containing elevated phosphate (2.6 mM), and the effects of FTI-277 on apoptosis were determined by assessing the levels of active caspase 3 after 2 and 4 hours (Fig 5A) and the number of apoptotic cells as a percentage of total cells after 12 hours (Fig 5B). These studies demonstrated that elevated phosphate increased the level of active caspase 3 and induced apoptosis in VSMC and these effects were significantly inhibited by co-incubation with FTI-277 (Fig 5). Furthermore, the Akt inhibitor, SH6, prevented the inhibition of phosphate-induced apoptosis by FTI-277 (Fig 5B), confirming that the increase in Akt phosphorylation with FTI-277 is crucial for its protective effects on VSMC.

### FTI-277 inhibits mineralization of aortic rings

We next investigated whether FTI-277 could inhibit mineralization of vessels which had been exposed to a uraemic environment for an extended period. For these experiments, we used aortic rings isolated from rats in end stage renal failure (equivalent to CKD stage 5) following sub-total nephrectomy. We chose to use this model as it provided an additional clinical context for our work. Renal function and systolic blood pressure were monitored in the rats every 2 weeks post-surgery. Progressive increases in systolic blood pressure to >150 mmHg and a urinary albumin:creatinine ratio > 1.0 mg/μmol were taken as indicative of a decline in renal function. Rats were sacrificed once end stage renal failure was reached (as indicated by rapid weight loss >20% body weight over 24–48 hours), which was approximately 7 months post-surgery. Terminal urine and plasma creatinine concentrations and blood urea nitrogen were recorded. These studies confirmed that the animals which had undergone sub-total nephrectomy had entered end stage renal disease (see S3 Table).

**Fig 6:**
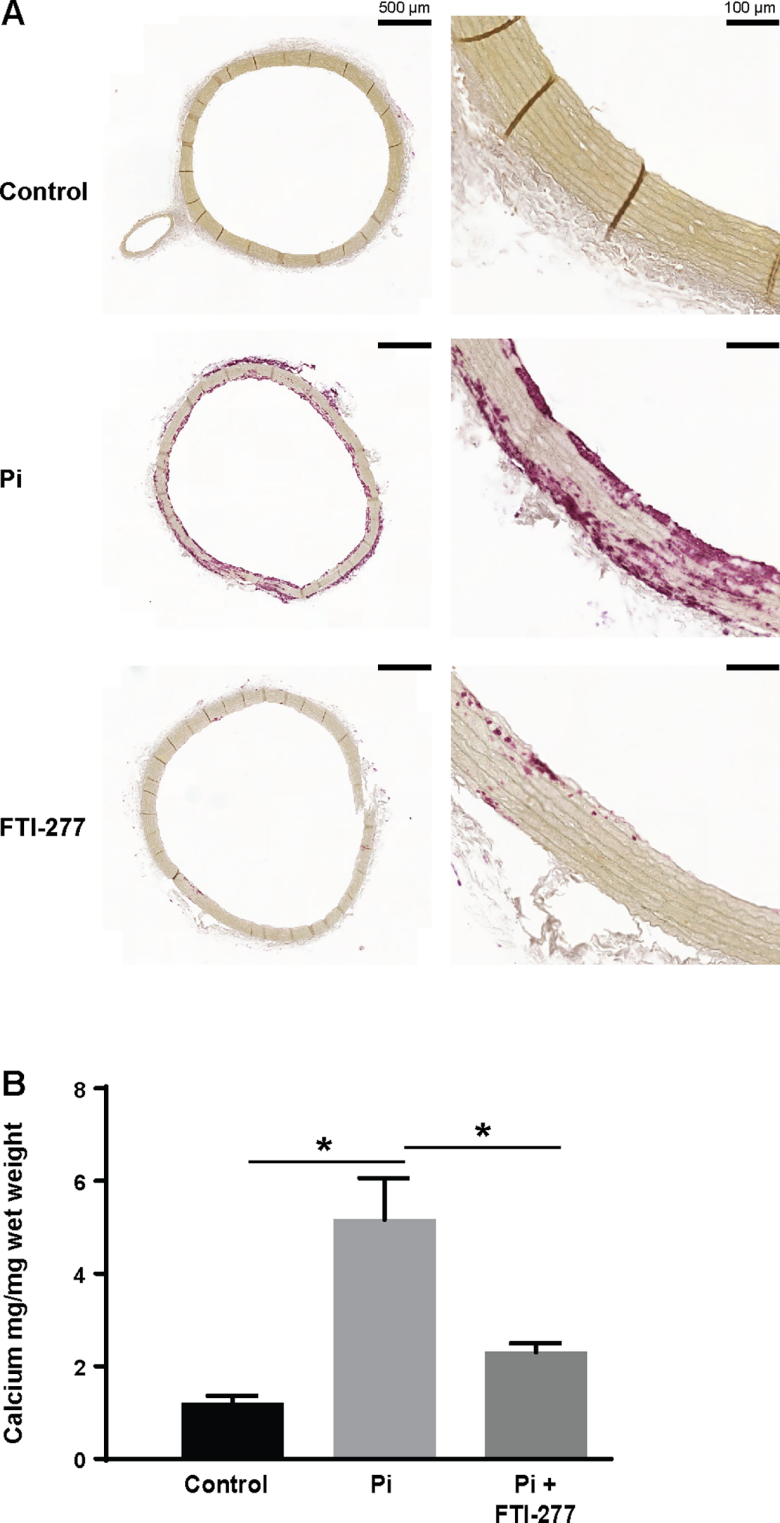
FTI-277 inhibits phosphate-induced mineralization of aortic rings from uraemic rats. Aortic rings from uraemic rats in end stage renal failure were incubated in control medium (Con; serum-free DMEM), phosphate medium (Pi; serum-free DMEM + 3.3 mM phosphate, 3.75 U/ml alkaline phosphatase), or phosphate medium + FTI-277 (10 μM) for 10 days. (**A**) Representative alizarin red stained sections of aortic rings from uraemic rats; bar = 500 μm (left panel) and 100 μm (right panel). (**B**) Quantification of mineralization in the aortic rings using the *O-*cresolphthalein complexone assay; n = 4 per group. Data are shown as mean ± SEM and were analyzed by one-way ANOVA and Tukey post-hoc tests; *p<0.05.

Therefore, the rats which had undergone sub-total nephrectomy and entered end stage renal disease were sacrificed and the ability of FTI-277 to inhibit phosphate-induced mineralization of aortic rings harvested from these animals investigated. Mineralization was assessed using histology (Fig 6A) and by measuring the calcium levels using the *O-*cresolphthalein complexone assay (Fig 6B). These studies showed mineralization was significantly increased when the aortic rings from uraemic rats were cultured in medium containing elevated phosphate and alkaline phosphatase, compared to control medium (Fig 6A and 6B). Furthermore, FTI-277 prevented this increase (Fig 6A and 6B).

## Discussion

We show for the first time that a farnesylation inhibitor, FTI-277, prevents *in vitro* mineral deposition by VSMC and *ex vivo* calcification of aortic rings isolated from uraemic rats. Furthermore, we demonstrate that FTI-277 may prevent *in vitro* mineralization by up-regulating PI3K/Akt signaling and inhibiting apoptosis, by preventing the osteogenic differentiation of VSMC and by elevating MGP expression, an inhibitor of calcification. In contrast, a second farnesyl transferase inhibitor, manumycin A, inhibits farnesylation, but does not activate Akt signaling or prevent mineralization. Together, these results demonstrate that activation of PI3K/Akt signaling by FTI-277 is crucial for its ability to attenuate vascular calcification. These results also extend previous studies by highlighting the potential of this drug to inhibit calcification in the absence of atherosclerosis, and in tissues and cells already primed to mineralize.

Our demonstration that FTI-277 inhibits mineralization by up-regulating PI3K/Akt signaling and inhibiting apoptosis is consistent with previous studies highlighting the importance of this pathway in preventing mineral deposition by VSMC [17, 43, 44]. Furthermore, the demonstration that manumycin A has no effect on either mineralization or Akt signaling in VSMC, even though Ras farnesylation is reduced, supports our suggestion that the induction of PI3K/Akt signaling in VSMC, rather than Ras inhibition *per se*, is crucial for the effects of FTI-277 on these cells. Previous studies have shown that Akt activation by FTI-277 can also occur in β-islet cells; Akt is also activated when the β sub-unit of farnesyltransferase is knocked-down using siRNA [27]. How FTI-277 up-regulates Akt signaling is VSMC (or other cells) is still not known, although the presence of a farnesylation-dependent G-protein that inhibits Akt signaling in β-islet cells was demonstrated [27]. Different FTIs have previously been shown to activate [27, 28, 34], attenuate [29–31] or have no effect [32–34] on Akt signaling, suggesting the effects of these drugs may be cell-type and context-dependent and may also depend on the nature of the FTI itself. Therefore, it will be important to take these differences into account when considering the potential of different FTIs as anti-calcification therapies.

The significance of inhibiting farnesylation for the inhibition of VSMC calcification by FTI-277 is not clear, as our results suggest that this drug inhibits calcification in a Ras-independent manner in VSMCs. FTIs can prevent the farnesylation of many other proteins, including prelamin A and mutant prelamin A [8, 12]. It is through this mechanism that tipifarnib and lonafarnib, respectively, are thought to alleviate cardiovascular disease in an animal model of Hutchinson-Gilford progeria syndrome [12] and to improve vascular stiffness and bone structure in children with this condition [13]. Farnesylated prelamin A has also been shown to accumulate in aged VSMC due to the decreased expression of the lamin A processing enzyme Zmpste24/FACE [46]. Aged and senescent VSMC have also been shown to mineralize at a faster rate than young cells [47–49] although whether this is due to accumulation of farnesylated prelamin A is unknown. Although we cannot exclude the possibility that FTI-277 modulates lamin A processing in our system, it is noteworthy that lamin A/C processing was not changed in uraemic ApoE-null mice treated with the FTI, R115777, even though calcification was inhibited [11].

Our results also demonstrate that FTI-277 prevents the osteogenic differentiation of VSMC whilst also increasing the expression of the mineralization inhibitor, MGP. These results are consistent with the demonstration that farnesylation is required for the osteogenic differentiation of mesenchymal stem cells, whereas geranylgeranylation is inhibitory [50]. Our results also support and extend a previous study showing decreased aortic Runx2 expression in uraemic ApoE-null mice treated with R115777 [11]. Furthermore, we also show that maintaining high levels of PI3K/Akt signaling in VSMC by over-expressing the constitutively active p110 sub-unit of PI3K, significantly increases MGP expression and inhibits mineralization. Together these results support the suggestion that FTI-277 reduces mineral deposition by VSMC by activating PI3K/Akt signaling which prevents their osteogenic differentiation and up-regulates the expression of MGP by these cells.

Akt signaling is a survival pathway in mammalian cells and increased apoptosis is a key factor in the pathogenesis of vascular calcification [17, 43, 44]. Here, we show that FTI-277 significantly inhibits phosphate-induced VSMC apoptosis and that these effects are blocked by co-incubation of cells with a selective Akt inhibitor, SH6. Although the differentiation and mineralization data were obtained following incubation of bovine VSMCs in calcification medium containing serum and βGP, whereas the apoptosis data were obtained following short-term incubation of human VSMCs in serum-free medium containing elevated phosphate, together these results suggest that FTI-277 may inhibit mineral deposition *in vitro* by activating PI3K/Akt signaling and preventing apoptosis.

Although further work is needed to identify the precise mechanism of action of FTI-277 in VMSC, our experiments have shown that this drug can ameliorate vascular calcification both *in vitro* and *ex vivo* in the absence of an atherosclerotic background. Recent clinical trials suggest that FTIs are well-tolerated in humans [16]. Therefore, these studies highlight the future potential of investigating the effects of FTIs in clinical vascular calcification, a condition associated with serious consequences, yet at present, out of reach of therapeutic intervention.

## Supporting information

S1 Fig FTI-277 and manumycin A inhibit Ras activation in VSMC. Confluent VSMCs were incubated in 10% FCS-DMEM ± FTI-277 (10 μM) or manumycin A (10 μM) for 77 hours, serum-starved for 2 hours and then stimulated with 10% FCS-DMEM for 5 minutes. Active Ras pull down assays were performed, and samples were analysed by western blotting using an anti-Ras antibody (top panel). The bottom panel shows western blots of cell lysates for total Ras. Data are representative of 3 experiments.
(TIF)

S2 Fig Manumycin A does not alter Akt phosphorylation or mineral deposition by VSMC. (**A**) Confluent VSMCs were incubated in 10% FCS-DMEM ± manumycin A (10 μM) or FTI-277 (10 μM) for 77 hours, serum-starved for 2 hours and then stimulated with 10% FCS-DMEM for 5 or 15 minutes. Cell lysates were analysed for phospho-Akt and total Akt expression using western blotting. Lanes 1, 4, 7 contain samples collected prior to serum stimulation (T = 0) and lanes 2, 5, 8 contain samples collected after 5 minutes stimulation; samples in lanes 3, 6, 9 were collected after 15 minutes stimulation. Lanes 4 and 5 (control samples) and lanes 7 and 8 (FTI-treated samples) are as shown in Fig 2A. (**B,C**) Confluent VSMCs were incubated in 10% FCS-DMEM containing βGP and DMSO (1:1000) (control, Con), or with 10% FCS-DMEM + βGP + manumycin A (10 μM, 20 μM). (**B**) Phase contrast images of alizarin red stained VSMCs on day 9; scale bar = 500 μm. (**C**) Mineralisation was quantified by dye elution (mean ± SEM; n = 7). Data were normalized using log_10_ and analyzed using one-way ANOVA and Tukey post-hoc tests. No significant differences were detected.
(TIF)

S1 Table Primer sequences for qPCR. (DOCX)

S2 Table Antibodies used in western blots. (DOCX)

S3 Table Data confirming end stage renal diseases in rats underdoing sub-total nephrectomy. Systolic blood pressure at 28 weeks post-surgery and terminal urine and plasma composition of SNx rats (n = 3) in end stage renal failure (equivalent to CKD stage 5 in humans) and age-matched sham rats (n = 3). Data are shown as the median and (interquartile range); statistical comparisons were not made due to small n numbers.
(DOCX)
