# Supplementary figures and images for: FTI-277 inhibits smooth muscle cell calcification by up-regulating PI3K/Akt signaling and inhibiting apoptosis
Source: PLoS One. 2018 Apr 24;13(4):e0196232. doi: 10.1371/journal.pone.0196232 (PMC5916518; doi:10.1371/journal.pone.0196232)

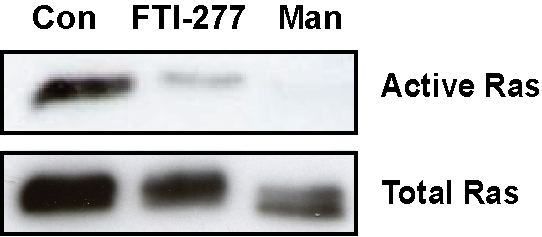

Supplement: S1 Fig — Confluent VSMCs were incubated in 10% FCS-DMEM ± FTI-277 (10 μM) or manumycin A (10 μM) for 77 hours, serum-starved for 2 hours and then stimulated with 10% FCS-DMEM for 5 minutes. Active Ras pull down assays were performed, and samples were analysed by western blotting using an anti-Ras antibody (top panel). The bottom panel shows western blots of cell lysates for total Ras. Data are representative of 3 experiments. (TIF) [file pone.0196232.s001.tif]

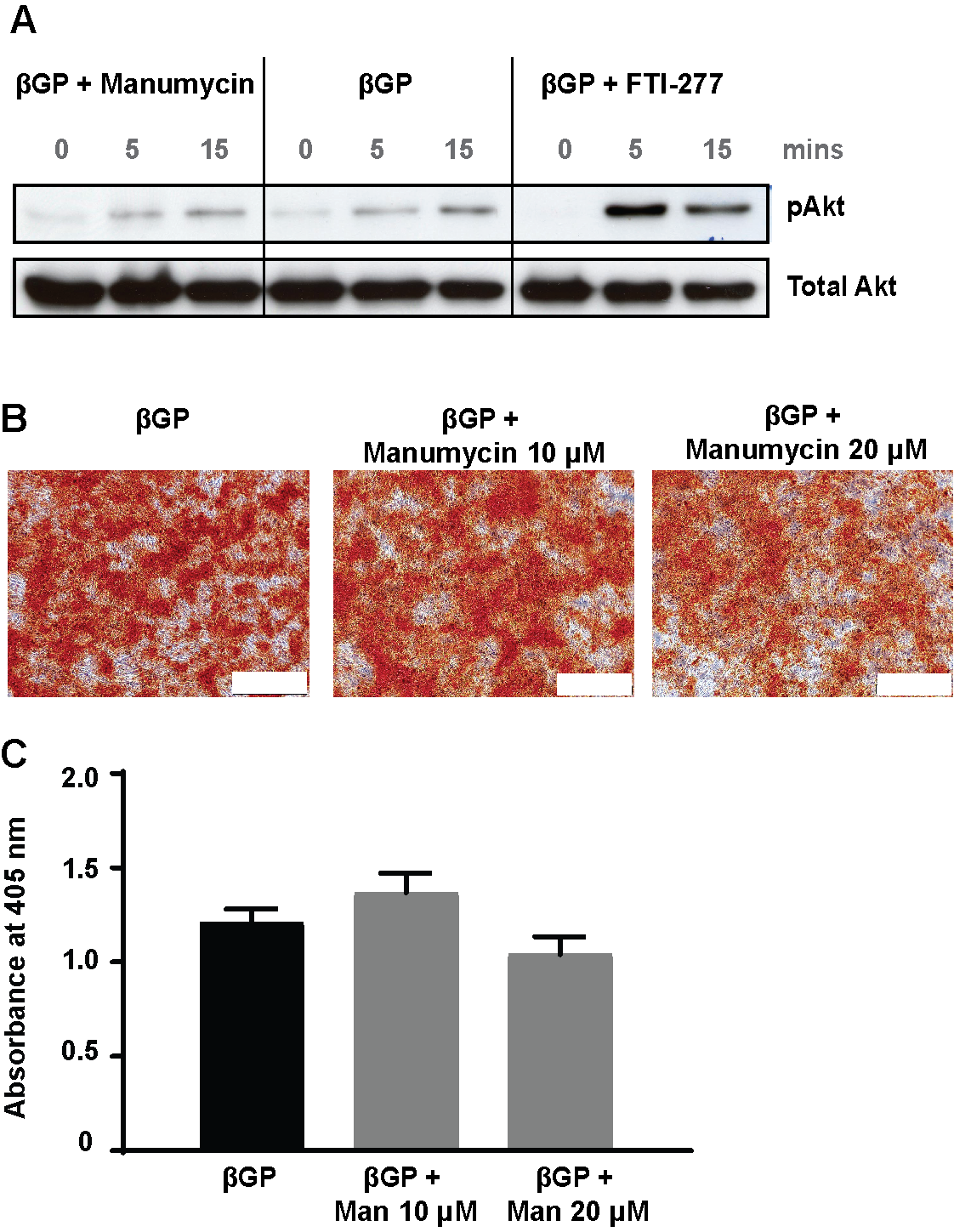

Supplement: S2 Fig — (A) Confluent VSMCs were incubated in 10% FCS-DMEM ± manumycin A (10 μM) or FTI-277 (10 μM) for 77 hours, serum-starved for 2 hours and then stimulated with 10% FCS-DMEM for 5 or 15 minutes. Cell lysates were analysed for phospho-Akt and total Akt expression using western blotting. Lanes 1, 4, 7 contain samples collected prior to serum stimulation (T = 0) and lanes 2, 5, 8 contain samples collected after 5 minutes stimulation; samples in lanes 3, 6, 9 were collected after 15 minutes stimulation. Lanes 4 and 5 (control samples) and lanes 7 and 8 (FTI-treated samples) are as shown in Fig 2A. (B,C) Confluent VSMCs were incubated in 10% FCS-DMEM containing βGP and DMSO (1:1000) (control, Con), or with 10% FCS-DMEM + βGP + manumycin A (10 μM, 20 μM). (B) Phase contrast images of alizarin red stained VSMCs on day 9; scale bar = 500 μm. (C) Mineralisation was quantified by dye elution (mean ± SEM; n = 7). Data were normalized using log10 and analyzed using one-way ANOVA and Tukey post-hoc tests. No significant differences were detected. (TIF) [file pone.0196232.s002.tif]
