# Supplementary material for: FTI-277 inhibits smooth muscle cell calcification by up-regulating PI3K/Akt signaling and inhibiting apoptosis
Source: PLoS One. 2018 Apr 24;13(4):e0196232. doi: 10.1371/journal.pone.0196232 (PMC5916518; doi:10.1371/journal.pone.0196232)
Supplement: S1 Table — (DOCX) [file pone.0196232.s003.docx]

**Supplemental Table S1: Primer sequences for qPCR**

| **Gene** | **Primers** |
| --- | --- |
| Runx2 | F: CCCAAGTTGCCACCTATCAC  R: TGAGGCGATCAGAGAACAAA |
| Msx2 | F: CTGGTCAAACCCTTCGAGAC  R: AGGAGCTGGGACGTGGTAAA |
| αSMA | F: ACCGCATGCAGAAGGAGA  R: GAGCCACCAATCCAGACAGA |
| MGP | F: AATAACGGTCGTAGGCAGCA  R: AGCCCAAGAGAGAATCCGAG |
