# Supplementary material for: FTI-277 inhibits smooth muscle cell calcification by up-regulating PI3K/Akt signaling and inhibiting apoptosis
Source: PLoS One. 2018 Apr 24;13(4):e0196232. doi: 10.1371/journal.pone.0196232 (PMC5916518; doi:10.1371/journal.pone.0196232)
Supplement: S2 Table — (DOCX) [file pone.0196232.s004.docx]

**Supplemental Table S2: Antibodies used in western blots**

| **Antibody** | **Details** | **Supplier** | **Block** | **Dilution** |
| --- | --- | --- | --- | --- |
| Akt | Polyclonal rabbit anti-Akt (#9272) RRID: AB_329827 | Cell Signaling | 5% (w/v) milk | 1:1000 |
| Phospho-Akt | Polyclonal rabbit anti-pAkt(Ser473) (#4060) RRID: AB_2315049 | Cell Signaling | 5% (w/v) milk | 1:1000 |
| Caspase 3 | Polyclonal rabbit anti-Caspase 3 (#9662) RRID: AB_331439 | Cell Signaling | 2% (v/v) fish gelatin | 1:500 |
| Cleaved caspase 3 | Polyclonal rabbit anti-Cleaved caspase3 (Asp175) (#9661) RRID: AB_2341188 | Cell Signaling | 2% (v/v) fish gelatin | 1:500 |
