# Supplementary material for: FTI-277 inhibits smooth muscle cell calcification by up-regulating PI3K/Akt signaling and inhibiting apoptosis
Source: PLoS One. 2018 Apr 24;13(4):e0196232. doi: 10.1371/journal.pone.0196232 (PMC5916518; doi:10.1371/journal.pone.0196232)
Supplement: S3 Table — Systolic blood pressure at 28 weeks post-surgery and terminal urine and plasma composition of SNx rats (n = 3) in end stage renal failure (equivalent to CKD stage 5 in humans) and age-matched sham rats (n = 3). Data are shown as the median and (interquartile range); statistical comparisons were not made due to small n numbers. (DOCX) [file pone.0196232.s005.docx]

**Supplemental Table S3**: **Data confirming end stage renal diseases in rats underdoing sub-total nephrectomy.** Systolic blood pressure at 28 weeks post-surgery and terminal urine and plasma composition of SNx rats (n = 3) in end stage renal failure (equivalent to CKD stage 5 in humans) and age-matched sham rats (n = 3). Data are shown as the median and (interquartile range); statistical comparisons were not made due to small n numbers.

|  | Systolic blood pressure  (mmHg) | Urine albumin:creatinine ratio  (mg/µmol) | Serum creatinine  (µmol/L) | Blood urea nitrogen (BUN)  (mmol/L) |
| --- | --- | --- | --- | --- |
| SNx | 163.8  (158.9-164.3) | 2.96  (1.55-3.55) | 263.4  (175.9-459.7) | 62.3  (59.7-74.8) |
| Sham | 130.3  (124.6-136.2) | 0.31  (0.06-0.54) | 76.0  (54.8-88.4) | 8.6  (6.2-12.2) |
